# Analytical Differentiable Finite-Resolution Density Map Calculation in CCTBX/Phenix

**DOI:** 10.1063/4.0000943

**Published:** 2025-10-27

**Authors:** Pavel Afonine, Paul Adams, Alexandre Urzhumtsev

**Affiliations:** 1 LBNL, Berkely, CA, USA; 2 IGBMC, Strasbourg, Illkirch, France

## Abstract

Beyond validation, publication, and other analyses, the final stage of structure determination using cryo-EM typically involves atomic model refinement against experimental data. This refinement is most naturally performed in real space - bypassing Fourier space - because all objects at this stage, including models and maps, exist in real space. However, many tools currently used in cryo-EM structure determination originate from and remain anchored to crystallography, which primarily operates in reciprocal (Fourier) space.

While Phenix tools specifically designed for cryo-EM during the resolution revolution were tailored to operate in real space - such as phenix.real_space_refine for atomic model refinement against maps, which accounts for 95% of structures deposited in the PDB using cryo-EM - they are still suboptimal in at least two aspects.

First, the refinement target for coordinate refinement in phenix.real_space_refine is perhaps the simplest and fastest to compute, as it focuses on fitting atoms to the nearest density peaks without considering the overall shape of the density map. The advantage of this approach is that it enables refinement of very large molecules on relatively modest computing resources (e.g., laptops). However, the drawback is the need for excessive geometric restraints to compensate for the simplicity of this refinement target, which does not account for the shape of the map.

The second limitation is that ADP (B-factor) and occupancy refinements still require a bypass through Fourier space, as they rely on spatial integration of density peaks for fitting.

Here, we will focus our discussion on implementing resolution-truncated density map calculations in CCTBX and Phenix using accurate, differentiable analytic approximations of density maps. This implementation will enable more accurate real-space refinement in Phenix for all atomic model parameters (coordinates, ADPs, occupancies, etc.), eliminating the need for Fourier space entirely in this process.

Additionally, making this approach available in the freely accessible CCTBX framework will provide the broader community with a uniform method for computing finite-resolution density maps and their derivatives with respect to atomic model parameters. This is particularly valuable for machine learning-based model building and refinement approaches, where algorithms for computing differentiable finite-resolution density maps are essential (e.g., qFit, ROCKET).

